# Increased elasticity of melanoma cells after low-LET proton beam due to actin cytoskeleton rearrangements

**DOI:** 10.1038/s41598-019-43453-7

**Published:** 2019-05-07

**Authors:** Katarzyna Jasińska-Konior, Olga Wiecheć, Michał Sarna, Agnieszka Panek, Jan Swakoń, Marta Michalik, Krystyna Urbańska, Martyna Elas

**Affiliations:** 1 Department of Biophysics, Faculty of Biochemistry, Biophysics and Biotechnology, Gronostajowa 7, Kraków, Poland; 2 Institute of Nuclear Physics, Polish Academy of Sciences, Radzikowskiego 152, Kraków, Poland; 3 Department of Cell Biology, Faculty of Biochemistry, Biophysics and Biotechnology, Gronostajowa 7, Kraków, Poland

**Keywords:** Atomic force microscopy, Cellular motility, Cell growth

## Abstract

Cellular response to non-lethal radiation stress include perturbations in DNA repair, angiogenesis, migration, and adhesion, among others. Low-LET proton beam radiation has been shown to induce somewhat different biological response than photon radiation. For example, we have shown that non-lethal doses of proton beam radiation inhibited migration of cells and that this effect persisted long-term. Here, we have examined cellular elasticity and actin cytoskeleton organization in BLM cutaneous melanoma and Mel270 uveal melanoma cells. Proton beam radiation increased cellular elasticity to a greater extent than X-rays and both types of radiation induced changes in actin cytoskeleton organization. Vimentin level increased in BLM cells after both types of radiation. Our data show that cell elasticity increased substantially after low-LET proton beam and persisted long after radiation. This may have significant consequences for the migratory properties of melanoma cells, as well as for the cell susceptibility to therapy.

## Introduction

Direct photon irradiation of a tumor from radioisotopes such as ^125^I or ^106^Ru is the main form of treatment in the management of uveal melanoma known as brachytherapy. It allows functional preservation of the eye in 52% of patients^1^, although it deteriorates in time and 10 years after therapy 68% of patients have poor visual acuity^2^. In the case of large tumors, or tumors located close to the optic nerve, proton beam irradiation may also be employed^3,4^. The main advantage of proton beam therapy over photon radiation is highly localized energy deposition at the end of protons range. Despite the fact that uveal melanoma is usually well controlled by radiation therapy, approximately 50% of patients develop metastases within 7 years of  ^5^ diagnosis. Uveal melanoma metastases are found primarily in the liver (90%), but also in lung (24%) and bone (16%). Within 2 years of developing metastatic disease, 70% of patients die, as there is no effective treatment^6–8^. Although radiation therapy has long been used in clinics^5^, little is known about the effect it has on key cellular properties such as cell elasticity.

Cellular elasticity is strongly connected to cancer invasion and migration during metastasis^9^, and its significance was recently demonstrated in the case of melanoma^10^. It was shown that melanoma cells that had higher Young modulus (less elastic) were less capable to penetrate different barriers than cells with lower Young modulus^11^ (more elastic), i.e. higher elasticity of a cell is related with increased invasiveness. Cell elasticity is strongly connected to the cytoskeleton of the cells and very little data focusing on the connection of cell elastic properties and their cytoskeleton after radiation, especially after proton beam radiation exist.

So far it has been reported that photon radiation, such as X-rays (5–20 Gy) caused visible reorganization of actin cytoskeleton, manifested as an increase of the peripheral actin fibers and stress fibers appearance^12^. A similar effect was also observed in the case of endothelial cells irradiated with X-rays, in which these modifications lead to lower motile activity of the cells^13^. An example study of the cell biomechanical properties performed on squamous carcinoma cells after photon radiation demonstrated that irradiated cells had higher elasticity than non-irradiated cells. This was linked to the alterations in the cytoskeleton organization^14^. Likewise, spatial reorganization of cytoskeleton was detected in endothelial cells in response to shear stress, even 12 hrs after exposure, manifesting in a larger number of thicker and longer stress fibers^15^. None of the studies reported longer time-scale changes.

Despite being claimed that both types of radiation generate comparable biological effects, there are several reports to the contrary, showing differences in cellular response exerted by photon or proton beam radiation, including DNA damage, cell cycle inhibition and cell migration among others^16–19^.

We have shown previously that sublethal doses of proton beam (low-LET), in contrast to photon irradiation slightly inhibited cellular movement in primary uveal melanoma cells and metastatic cutaneous melanoma cells^18^. Here we show that the reported differences in cellular motility may result from alterations in the cell cytoskeleton organization and corresponding mechanical properties of the cells that persist long-term after irradiation. Biomechanical analysis, as well as F-actin and vimentin staining have shown that both low-LET proton beam and X-rays induced higher elasticity due to perturbed cytoskeleton in melanoma cells.

## Results

### Irradiation inhibits cell proliferation long-term

**Figure 1:**
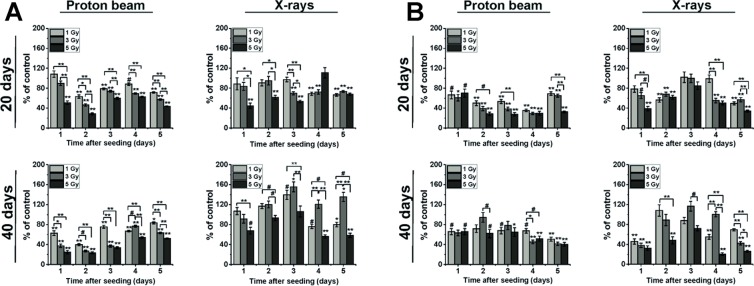
Cell counting of Mel270 (**A**) and BLM (**B**) cells performed 20 and 40 days after treatment with proton beam radiation or X-rays. Cells were irradiated, cultured at standard conditions and then seeded at 20 and 40 days post-treatment and the assay was conducted during five next days. Experiment was performed in triplicate and for every repetition, cells from 4 wells were counted. Results are presented as the percent of the untreated control. Mean values ± SEM. ^#^p < 0.05; *p < 0.01; **p < 0.001.

Cell proliferation was checked immediately after irradiation, and as expected, a substantial inhibition of cell growth was observed after both types of radiation, with low-LET proton beam eliminating more highly-proliferating colonies than X-rays^18^. At longer times after treatment, i.e. 20 and 40 days both Mel270 and BLM cells still exhibited decreased proliferation rate at 30–70% of control after low-LET proton beam (Fig. 1). Cells treated with X-rays displayed similar tendency at 20 days, but at 40 days post-treatment a stimulation of Mel270 proliferation was observed with its maximum at 3^rd^ day after seeding, reaching 139% and 155% for groups irradiated with 1 and 3 Gy, respectively. The impact of X-rays was subtler in BLM cells, with slight inhibition seen at 20 days, and variations around the control at 40 days (Fig. 1). These results confirm that cellular response to low doses of radiation is seen long-term after the treatment in both Mel270 and BLM cells.

### Slower Mel270 and BLM cell migration after proton beam

**Figure 2:**
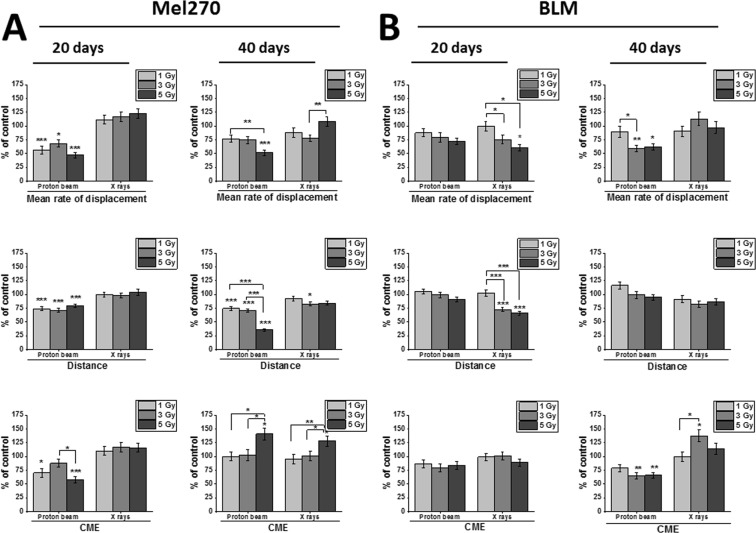
Cellular migration properties of Mel270 (**A**) and BLM (**B**) cells treated with proton beam radiation or X rays. Individual cell movements were evaluated at 20 and 40 days after irradiation with either proton beam or X-rays and three parameters were calculated: ‘Mean rate of displacement’, i.e. the distance from the starting point direct to the cell's final position/time of recording; ‘Distance’, i.e. the total cell trajectory (μm) and CME (coefficient of movement efficiency), i.e. the ratio of cell displacement to the cell trajectory length. Mean values presented as percent of control; *p < 0.05, **p < 0.01, ***p < 0.001. Data partially presented in Jasinska *et al*.^18^.

Single cell migration was determined at 20 and 40 days after radiation by tracking individual cells using time-lapse microscopy. Proton beam irradiation inhibited multiple parameters of Mel270 migration both at 20 and 40 days after treatment (Fig. 2A), whereas X-rays had very little effect. In contrast, irradiation of BLM cells resulted in migration inhibition after X rays at 20 days, and after proton beam at 40 days post-treatment (Fig. 2B).

### Cells are much more elastic after low-LET proton beam

**Figure 3:**
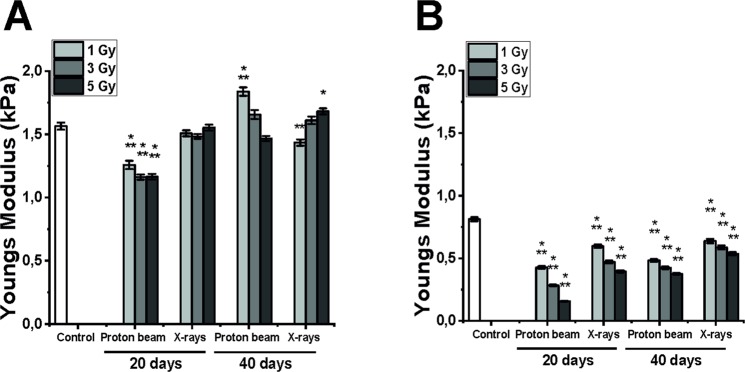
Impact of proton beam radiation and X-rays on the elastic properties of Mel270 (**A**) and BLM cells (**B**). Mean of Young modulus measured from 325 cells ± SEM values; *p < 0.05; **p < 0.01; ***p < 0.001, against untreated control.

**Figure 4:**
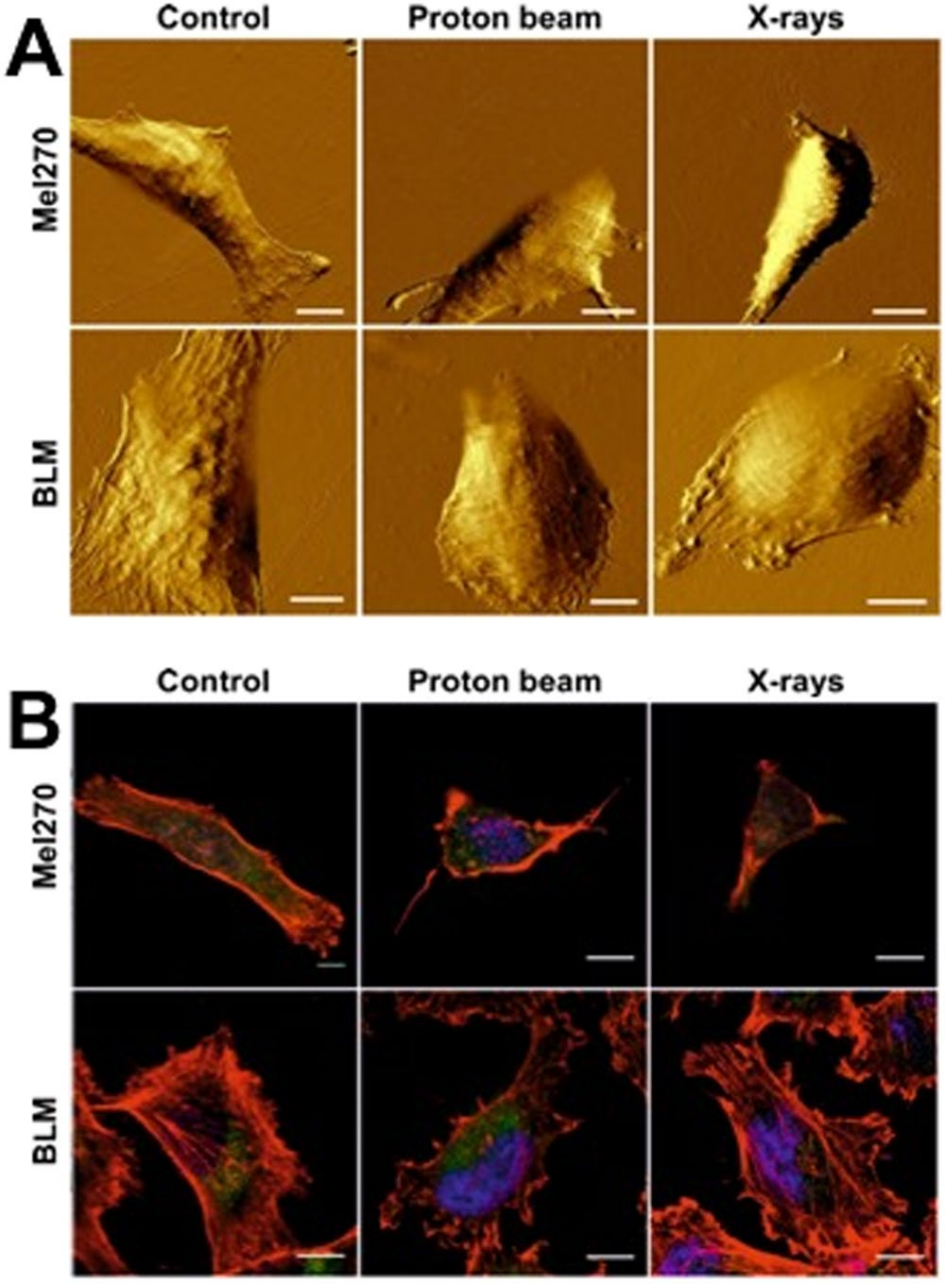
(**A**) AFM amplitude images of the BLM and Mel270 cells. (**B**) Confocal microscopy images of the same cells stained for actin (red), vimentin (green) and nucleus (blue). Scale bars in all images represent 10 um. Cells were treated with 3 Gy of either proton beam or X-rays and cultured for 20 days.

Cellular elasticity was determined at 20 and 40 days post-irradiation. Untreated Mel270 and BLM cells exhibited a normal distribution of the Young’s modulus values, although the mean value of Mel270 cells was almost two times higher than that of BLM cells (1.5 vs 0.8 kPa, respectively, Fig. 3A,B). This shows that Mel270 are much stiffer than BLM cells and confirms the widely accepted paradigm that metastatic cells are more elastic than cells from the primary tumor. In general, a substantial decrease of the Young’s modulus values was observed in both cell lines after low-LET proton beam. For the highest dose of the low-LET proton beam, Mel270 were approximately 1.5-times more elastic and BLM 5 times more elastic than control (at 20 days post-radiation). In the case of X-rays and proton beam at 40 days the elasticity of Mel270 was not changed significantly. On the other hand, BLM cells displayed a noticeable decrease in the Young’s modulus values at both 20 and 40 days after X-ray radiation. The histograms (Suppl. Fig. A1) show more random distribution at 40 days post-treatment than at 20, especially for Mel270 cells. BLM cells at 20 days after low-LET proton beam exhibit very narrow distributions, especially for the dose of 5 Gy. Moreover, morphological changes could be observed by AFM imaging in the examined cells (Fig. 4). As evident from the images, Mel270 after both types of radiation became more constrict with no cytoskeleton features being pronounced. On the other hand, in BLM cells after proton irradiation less actin fibers were present in the center region of the cell, whereas in BLM cells after X-rays thick marginal actin fibers were visible. Furthermore, BLM cells displayed more fibroblastoid-like phenotype with higher number of internal actin fibers, known as stress fibers, combined with numerous protrusions being visible (Fig. 4B).

### Alterations in marginal and internal actin fiber thickness following radiation

**Figure 5:**
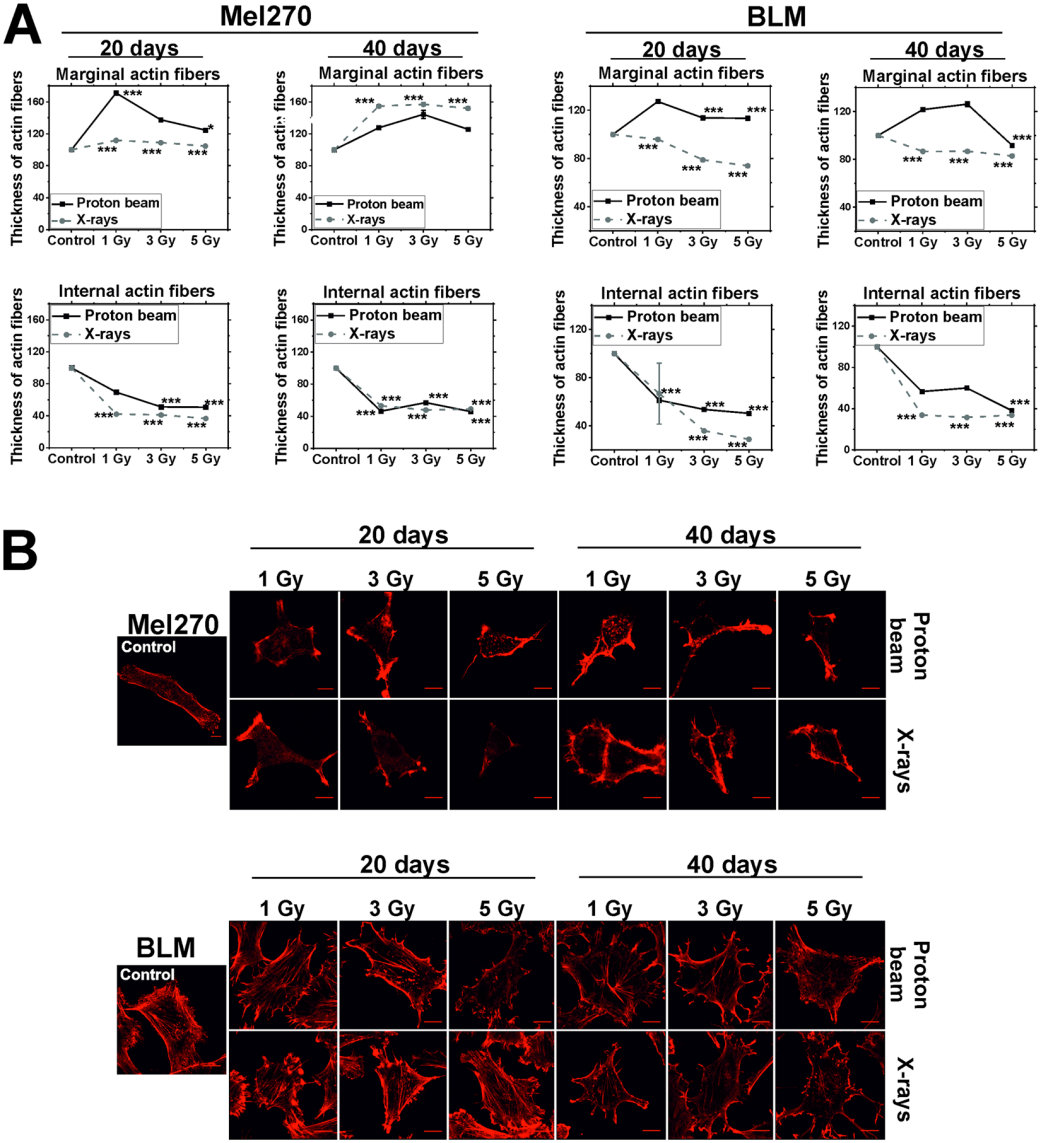
(**A**) Thickness of actin fibers in Mel270 and BLM cells treated with low-LET proton beam radiation or X-rays. Measurements of 15 cells in each experimental group were taken at 20 and 40 days following radiation. Two types of fibers were measured: marginal and internal also called stress fibers with 15 measurements for each type of fibers per cell. Results are presented as the percent of the untreated control. Mean values ± SEM. *p < 0.05; **p < 0.01; ***p < 0.001. (**B**) Representative images of actin cytoskeleton stained in Mel270 and BLM cells after treatment with low-LET proton beam radiation or X-rays. Cells were stained with phalloidin conjugated with Alexa 546. Scale bar: 10 μm.

Analysis of actin cytoskeleton revealed disintegration of internal actin fibers in both types of cells (Fig. 5A,B). The thickness of internal actin fibers decreased in all groups in both cell lines at 20 and 40 days, with values in the range of 36–69% of the control level. The change in thickness of marginal actin fibers following radiation treatment varied based on cell line treated. In Mel270 at 20 days only proton beam leads to increase in thickness (with maximum value of 171% of control for 1 Gy), and at 40 days the effect is similar for both types of radiation (Fig. 5A, left). In BLM cells there was an increase in thickness of marginal fibers after proton beam, and a decrease after X rays (Fig. 5A, right). In summary, the effect on internal fibers was similar for both types of radiation and the effect of proton beam on marginal fibers was more pronounced. Actin aggregates were also seen in Mel270 cells (Fig. 4B). Taken together, these changes mark substantial alterations in the cytoskeleton structure.

### Increased vimentin level in cutaneous melanoma after radiation

**Figure 6:**
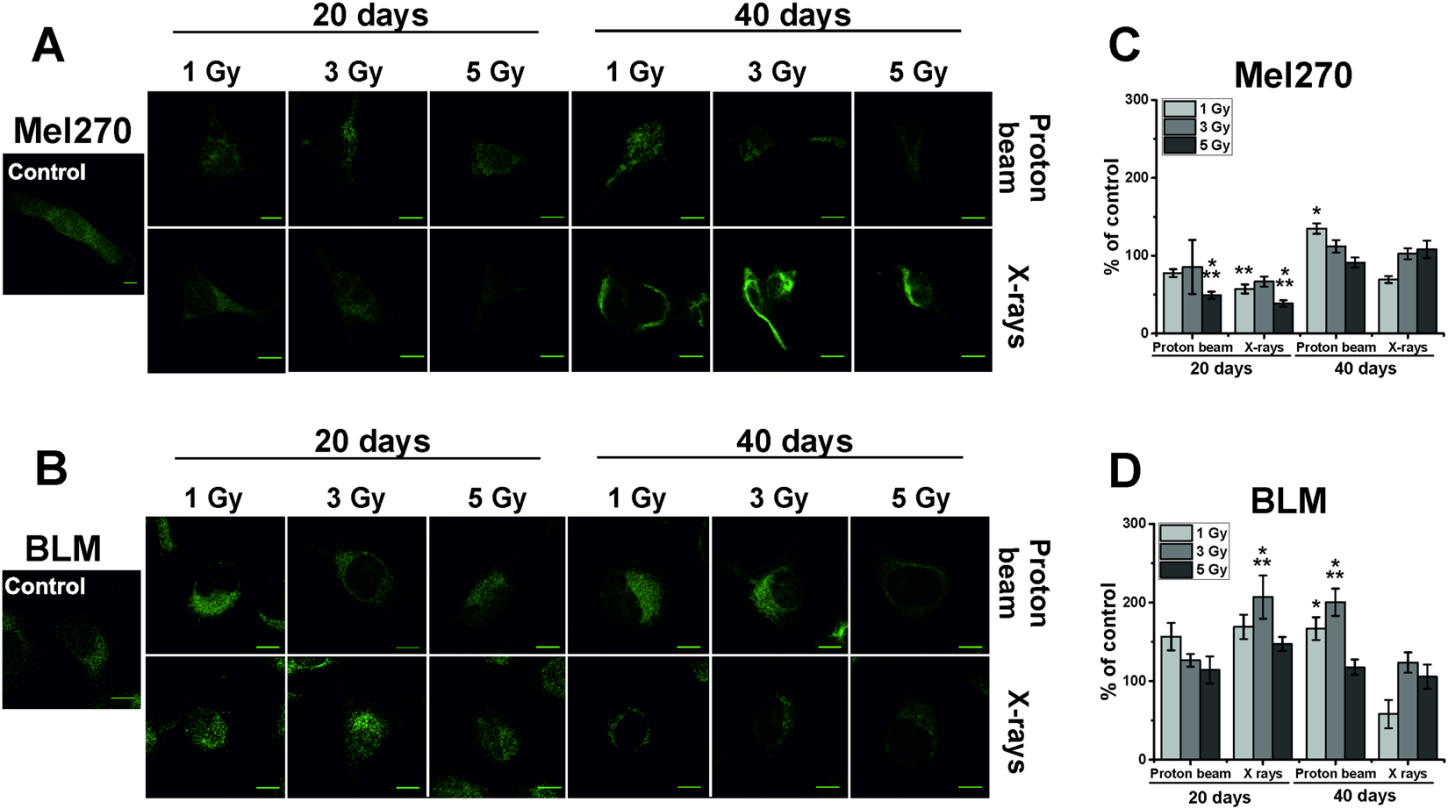
Representative images of vimentin stained in Mel270 (**A**) and BLM (**B**) cells after treatment with proton beam radiation or X-rays. The corrected total cell fluorescence (CTCF) was measured in Mel270 (**C**) and BLM (**D**) cells. For each experimental group 15 cells were measured. Results are presented as the percent of the untreated control. Mean values ± SEM. *p < 0.05; **p < 0.01; ***p < 0.001. Scale bar: 10 µm.

Vimentin is an intermediate filament that maintains cell and tissue integrity and confers resistance to mechanical stress^20^, but also have complex regulatory functions^21^. In order to determine the level of vimentin in the cells, the corrected total cell fluorescence (CTCF) was calculated from confocal microscopy images of cells stained for vimentin (Fig. 6). In Mel270 cells, a decrease of vimentin was seen at 20 days post-treatment with the lowest values of 49% and 38% after 5 Gy of the low-LET proton beam and X-rays, respectively. At 40 days there was no change, except for an increase of up to 134% for 1 Gy of the proton beam. However, in BLM cells an increased level of vimentin was seen, especially at 40 days after proton beam and 20 days after X-rays. BLM cells treated with 3 Gy of X-rays displayed an increase of the level of this protein to 206% of control. At 40 days we observed values above the control only in the groups treated with 1 or 3 Gy of the low-LET proton beam. These results correspond well with our previous analysis of vimentin levels performed by western blot analysis^18^.

### Adhesion and migration proteins

**Figure 7:**
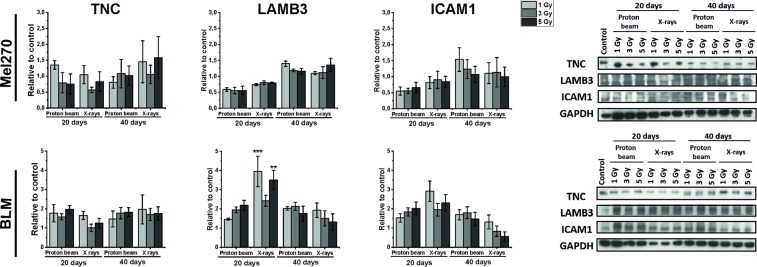
TNC, LAMB3 and ICAM1 protein expression determined with Western Blot in Mel270 and BLM cells after treatment with different doses (1, 3, 5 Gy) of proton beam or X rays. Cells were lysed 20 and 40 days after irradiation. Protein bands were densitrometrically analyzed and adjusted against GAPDH. Results represent average of 3 independent experiments. Mean ± SEM. *p < 0.05; **p < 0.01; ***p < 0.001. Samples were analyzed together as indicated by the frames (only TNC control was taken from another blot, see Suppl. Fig. A3).

Radiation did not change the level of three adhesion and migration proteins studied: TNC, LAMB3 and ICAM1, except for an increase seen in the level of LAMB3 in BLM cells 20 days after X-rays. Decreasing trends in LABM3 and ICAM1 in Mel270 cells were not statistically significant (Fig. 7).

## Discussion

In this work we have examined two types of melanoma cells: uveal melanoma Mel270 and cutaneous melanoma BLM cells, differing in their genetic phenotype. Cutaneous melanoma is generally not managed with radiation, except for lentigo malignant melanoma or as a palliative treatment^22^. Uveal melanoma, on the other hand, is routinely treated with both photon brachytherapy and low-LET proton beam radiation^5,8,23,24^. Since both types of melanoma metastasize, during which the cells undergo significant deformation of their body; the influence of radiation on their mechanical properties and thus their metastatic capabilities had to be addressed.

In our study, untreated BLM cells, originating from a metastasis of cutaneous melanoma, were twice more elastic than uveal Mel270 cells, originating from a primary uveal tumor. Irradiation led to an increased elasticity – in BLM cells after both types of radiation, whereas in Mel270 cells only after low-LET proton beam radiation. It has been reported that modifications of the actin cytoskeleton affect the mechanical properties of the cells to the highest extent^25–30^, therefore, in this study we have focused primarily on this cytoskeleton component.

Even though the total levels of actin were not changed after irradiation, actin cytoskeleton rearrangements were detectable long-term after the treatment. Significant decrease in thickness of inner actin stress fibers in all treated groups and an increase in the thickness of marginal fibers in all proton-beam treated groups was observed. The internal actin fibers seem to be more relevant to cellular elasticity^9,31^. Both Young modulus decrease with dose as well as migration parameters decrease with dose was accompanied with a decrease in thickness of internal fibers. These parallel changes were seen in Mel270 cells after proton beam radiation, and in BLM cells for both types of radiation (see Suppl. Fig. A2). Therefore, we concluded that the observed actin rearrangements may be partially responsible for higher elasticity in the treated cells. Of course, it should be noted that other cell constituents that build up the cytoskeleton of a cell such as intermediate filaments and microtubules could also be affected by radiation. However, their contribution to cell mechanics is less important than that of actin cytoskeleton^32^. Cellular elasticity is an end-result of many factors, including the length and thickness of the stress fibers, which are anchored to the adhesion focal points, as well as the number of internal fibers/arcs in the subcortical part of the cell^33^. Moreover, higher vimentin level in BLM cells might also play a role, as vimentin has been shown to enhance cellular elasticity^20^. Further elucidation of the mechanism of cellular softening after low-LET proton beam radiation might be provided by studying the changes in the expression of vinculin, myosin and microtubules organization of irradiated cells.

The increase in cell deformability directly correlates with progression from normal to transformed malignant cell^34^, as well as towards more metastatic phenotype^25,35,36^. Higher elasticity of cells is linked to weaker actin filament polarization and therefore leads to lower migration abilities^37^. Furthermore, migrating cells generally have thicker stress fibers than non-motile cells^37^ and thicker stress fibers correspond with higher values of the Young’s modulus of cells^30^. Mechanical softening of cancer cells and modification of their adhesion to extracellular matrix increased their capacity to escape the primary tumor^38^. It is worth pointing out that a substantial increase in elasticity might be connected to loss of membrane viability and cellular well-being, however, in our experiments cells were viable and proliferating.

Higher elasticity is thought to ease invasion and migration^11,28^. It should be noted that elasticity is a general parameter describing cell mechanical properties, which results from different factors such as: cytoskeleton organization, in particular actin organization, osmotic pressure, adhesion, etc^31^. Indeed, higher elasticity of a cell is related with increased invasiveness, because it is thought that the softer and more deformable a cancer cell is, the more likely it will invade different tissue barriers^11,25,39^. Accordingly, number of studies have shown that cells with similar migration capabilities but differing in their elasticity had different invasion capabilities^35^. However, in our study, we show that changes in the elasticity of the cells were accompanied by the disruption of actin cytoskeleton. Taking into consideration that actin cytoskeleton is one of the most important components of a cell for migration, it is very unlikely that cells could become more invasive when they have disrupted cytoskeleton. On the contrary, one may speculate that such substantial decrease in cellular stiffness as seen in BLM cells (up to 5 times) may completely unable cellular movement and result in decreased migratory properties.

The effect of X-rays on elasticity and migratory properties was very much cell line dependent, i.e. the elasticity and migration of Mel270 cells were only slightly affected. In contrast, we observed a decrease in the Young’s modulus values in BLM cells after X-rays (Fig. 3), and slight changes in migration potential of BLM cells after X-rays (Fig. 2). This points out that the same range of doses leads to slightly different effects in the tested cell lines. In general, the changes in cellular elasticity in Mel270 choroidal melanoma were much less pronounced. This may stem out from the fact that Mel270 cells origin from the primary tumor, and BLM from a metastasis of a cutaneous melanoma. Along with higher cell elasticity, BLM cells also exhibited a higher level of vimentin (after X-rays at 20 days and low-LET proton beam at 40 days), which may suggest the possibility of EMT transition switching phenotype in melanoma cells post-radiation. However, we have not detected any N-cadherin in the cells, which would be a confirmation of the switching phenotype (data not shown). Moreover, the effect of irradiation on the tumor microenvironment and interactions between cancer and other types of cells, should be further studied in this context, as it affects cellular elasticity and migratory properties as well^40^.

Radiation is known to enrich cellular population with cancer stem-like cells. The changes that we have observed at 20 and 40 days after radiation might represent an average over several cell subpopulations. We have shown previously the existence of three distinct cell populations in the untreated BLM and Mel270 cells, with a shift towards less-proliferative populations after irradiation. Therefore, the observed effects might be even higher if isolated subpopulations were studied. Other authors showed that the Young’s modulus of tongue carcinoma cells was decreased in a dose-dependent manner in 24 hrs after X-rays (1–4 Gy) with over 3-fold decrease for the highest dose, with accompanying decrease in actin filaments and increased migration^14^. The increased thickness of peripheral actin fibers was also shown in endothelial cells in response to photon radiation^12^. Other authors suggest that the effects of radiation undergo intensive time changes up to 7 days post-radiation^41^. However, long term effects that are more suitable in terms of a life span of a patient have not been described in literature yet, and thus were examined in this study.

We have checked the level of three proteins involved in adhesion and migration, TNC, LAMB3 and ICAM1, but did not found any significant changes in the protein level, except for an increase in LAMB3 20 days after X-radiation. Our previous proteomic study revealed increase in several proteins involved in migration and metastasis, i.e. moesin (actin remodelling, motility), actinin 4 (migration and metastasis), FAB-2 (migration, microtubule destabilizer), lamine A/C (PI3K/AKT/PTEN, adhesion, motility), lamine B (motility)^42^. In the same study we also observed a decrease in annexin 7 (motility) and vimentin. The latter might result from post-translational modifications, as here we have seen a clear increase in the vimentin level (Fig. 6).

Although the exact mechanisms of the observed elasticity and cytoskeleton changes are not fully clear, there are some indicators in the literature, which prompt further research. Several actin-associated proteins have been implicated in cytoskeleton changes after radiation. For example, ankyrin-1 (ANK1) alters the structure of the actin cytoskeleton and sustains limited cell migration during DNA damage^43^. Similar alterations in the arrangement of actin microfilaments and detachments from junctions were seen in HeLa cells exposed to IR and to ROS^44^. Such actin filaments rearrangements might be induced by RhoA, as rapid formation of actin filaments accompanied by redistribution of VE-cadherin adherent junctions in microvascular endothelial cells after radiation was mediated by RhoA/ROCK^12^.

It was shown that GNAQ mutation, one of the driver mutation in uveal melanoma, is present in Mel270 cells^45^. GNAQ mutation induces viability and migration of uveal melanoma cells via Notch signaling activation, which is mediated by YAP dephosphorylation and nuclear translocation^46^. Gαq activates YAP by acting on a Hippo-independent signaling network initiated by actin polymerization. Disruption of the actin cytoskeleton, e.g. due to ROS generated by radiation, diminishes both the basal activity of YAP and YAP hyperactivation^47^.

Interestingly, clinical data gathered from patients with uveal melanoma, do not indicate any significant difference in the mortality rate between radiation-treated patients and patients that undergone surgery^48,49^. This may suggest that irradiation of the tumor in the eye does not affect the process of metastasis. However, most authors would argue that metastases are seeded before the treatment or even before the diagnosis and remain dormant for many years^50^. Still, radiation may influence the cells seeding from the tumor after the treatment. Low, non-lethal doses tested may be relevant in the margin of the tumor treated with radiation.

A low-LET proton beam and photon radiation led to considerable rearrangements in the cytoskeleton of cells, affecting strongly their mechanical properties. Cellular elasticity increased more after proton beam irradiation. These changes were persistent long-term after the treatment and may be connected to an inhibition in cellular migratory properties. Taken together, these results suggest that low-LET proton beam radiation might have inhibitory effects on the migratory properties of melanoma cells. The exact molecular mechanism and signaling pathways leading to these effects require further studies.

## Methods

### Cell culture

Two melanoma cell lines were used: Mel270 - a primary human uveal melanoma cell line^51,52^, and metastatic BLM cells, derived from the lung metastases of skin melanoma^52^. Cells were cultured at 37 °C, 5% CO_2_ in RPMI media (Sigma-Aldrich, St. Louis, MO). Media were supplemented with 10% fetal bovine serum (Biological Industries, Cromwell, CT) and penicillin/streptomycin (Polpharma, Poland). The Mel270 cells were a gift from prof. M. Jager from Leiden University (The Netherlands) and BLM cells from Dr. G.N.P. van Muijen, Department of Pathology, Radboud University Nijmegen Medical Centre, Nijmegen (The Netherlands).

### Irradiation

Cell irradiation was performed at the Institute of Nuclear Physics, Polish Academy of Sciences (IFJ PAN), Cracow, Poland. For X-ray irradiation, Phillips MCN-323 tube operating at a voltage of 250 kVp and the dose rate of 1.8 Gy/min was applied. Proton beam irradiation took place at the Cyclotron Centre Bronowice at IFJ PAN. The 230 MeV proton beam was produced at the IBA Proteus C-235 cyclotron^53^. During the irradiation, the doses 1, 3, or 5 Gy have been delivered with a dose rate of 1 Gy/min, 2 Gy/min, and 6.6 Gy/min, respectively. At the center of cell container position i.e. at the depths 15.8 mm of the SOBP, the calculated Continues Slowing Down Approximation (CSDA) dose averaged LET_d_ was 2.8 keV/µm. A detailed description of the irradiation and dosimetry can be found elsewhere^18^. Doses between 1 and 5 Gy were chosen as they allowed long term post-treatment studies.

### Cell counting

The cells were seeded into 24-well plates (10^4^ cells per well), and cell numbers were determined at each consecutive day during the five days duration at 20 days (4th passage) and 40 days (7th passage) following radiation. Cells were trypsinized and counted using Bürker hemocytometer. The experiment was conducted three times and for each repetition, cells were counted from 4 wells.

### Cell migration

Time-lapse monitoring of individual cell movements was used as an indicator of cellular migration properties. The individual trajectories of cells were assessed 20 days and 40 days after irradiation in both Mel270 and BLM cells. Cells were plated at a density of 72 cells/mm^2^. After 48 hours the migration of cells was recorded at 37 °C for 10 h, at 10 min intervals. The trajectories of individual cells were evaluated from the changes in cell centroid location, as described previously^54^. For each cell, the following variables were determined^55^: (i) distance - the total length of the cell trajectory (µm), (ii) the total length of cell displacement (µm), i.e. the distance from the starting point direct to the cell’s final position; (iii) mean rate of displacement (μm/h), i.e. the distance from the starting point direct to the cell’s final position/time of recording; (iv) the mean speed of cell movement, i.e. the total length of cell trajectory/time of recording. The value of CME (Coefficient of Movement Efficiency) was calculated as the ratio of the total cell displacement to the total length of cell trajectory. For each value, 50 cells were analyzed from 3 different wells.

### Immunofluorescence staining and images analysis

Cells were plated on to microscope slides (Menzel-Glaser, Germany) in 3 cm dish (TPP, Switzerland) at a density of 15 × 10^4^ cells. After 24 hours, the cells were fixed with 4% paraformaldehyde (Sigma-Aldrich, USA) at room temperature. Permeabilization and blocking steps were performed using 0.1% Triton X-100 (Avantor Performance Materials, Poland) and 3% bovine serum albumin (Sigma-Aldrich, USA), respectively. The primary antibodies used for staining were Vimentin (D21H3) Rabbit mAbs (Cell Signaling Technology, USA) in 0.1% BSA at a concentration 1:300. In the second step, a secondary solution in 0.1% BSA was used which contained Alexa Fluor® 546 phalloidin (Thermo Fisher Scientific, USA), Alexa Fluor® 488 goat anti-rabbit IgG (H + L) (Thermo Fisher Scientific, USA) and Hoechst 33258 (Thermo Fisher Scientific, USA). The cells were incubated at room temperature in the dark. Glass cover slides were placed on basic microscope slides (Elektromed, Poland) using fluorescent mounting medium (Dako, USA). Samples were kept in dark at 4 °C until analyzed with a scanning laser confocal microscope (Zeiss LSM 880 with Airyscan). The experiment was done at two time-points, 20 and 40 days after irradiation.

The corrected total cell fluorescence (CTCF) was measured using ImageJ v.1.43U (Wayne Rasband, National Institute of Health, USA). Fluorescence intensity was measured from maximum intensity projection of the stack images according to the formula^56^: CTCF = Integrated Density − (Area of selected cell × Mean fluorescence of background readings). For each group, 15 cells were analyzed. Thickness of actin fibers in the cells was determined based on fluorescent images of the cells with stained F-actin using ImageJ v.1.43U (Wayne Rasband, National Institute of Health, USA). Two types of fibers were determined: marginal fibers and internal fibers also called stress fibers. From each experimental group, including the untreated control, the fibers of 15 cells were quantified with 15 measurements for each type of fibers per cell.

### Atomic force microscopy

Atomic force microscopy (AFM) analysis of the cells was conducted using a Bruker BioScope Catalyst AFM coupled with an inverted optical microscope (Axio Observer Z1 from Zeiss). Measurements were performed on cells maintained in culture medium at 37 °C. Mechanical analysis of the cells was made in force spectroscopy mode. Before the measurements, the AFM probe was positioned on top of an individual cell and aligned at the center of the cell body using bright field optical microscopy live view at ×400 magnification. Once aligned, force curves from a grid of 5 × 5 points were collected at a rate of 1 Hz. 15 cells per each condition were analyzed. Mechanical measurements were conducted using soft cantilevers with a nominal tip radius of 20 nm and spring constant of 0.01 N/m. For precise mechanical characterization, spring constants of the used cantilevers were routinely determined based on the thermal tune procedure^57^. Analysis of force curves was made using AtomicJ software^58^. In brief, the collected force-displacement curves were first converted into force-indentation curves and fitted with the Sneddon model. A detailed description of the mechanical analysis used in this work can be found elsewhere^59^.

### Western blot

Cell monolayers were lysed in RIPA buffer (Thermo Fisher Scientific) protease inhibitor cocktail (Roche, Switzerland), PMSF and sodium orthovanadate. The amount of protein was measured using the BCA kit (Sigma-Aldrich, St. Louis, MO) and stored at −80 °C until used. Equal amounts of protein (20 μg) were run on Bolt1Bis-Tris Plus gels (Thermo Fisher Scientific) and transferred to a nitrocellulose membrane. The membranes were blocked with 5% skim milk or in 5% BSA in a TBS buffer with 1% of Tween 20 for 1 h and incubated with primary antibodies against ICAM-1 and Tenascin C (D16C4) (Cell Signaling Technology, MA, USA), LAMB3 (CL3363) (Thermo Fisher Scientific) at 4 °C overnight. Membranes were washed 3 times in TBS and incubated with suitable secondary antibodies and then washed 3 times in TBS. Signals were detected using SuperSignal West Pico PLUS Chemiluminescent Substrate (Thermo Fisher Scientific).

### Statistical analysis

Statistical analysis was carried out using Statistica v12 (StatSoft. Inc.). Due to the comparison of more than three experimental groups, statistical significance was estimated using evaluation of homogeneity of variances with Levene’s Test followed by one-way analysis of variance (ANOVA). The differences were considered to be statistically significant at probability levels of p < 0.05, p < 0.01 and p < 0.001.

## Supplementary information

Dataset 1

## Data Availability

The datasets generated and analysed during the current study are available from the corresponding author on reasonable request.
