# Supplementary material for: Increased elasticity of melanoma cells after low-LET proton beam due to actin cytoskeleton rearrangements
Source: Sci Rep. 2019 May 7;9:7008. doi: 10.1038/s41598-019-43453-7 (PMC6504917; doi:10.1038/s41598-019-43453-7)
Supplement: Supplementary file 1 — Dataset 1 [file 41598_2019_43453_MOESM1_ESM.pdf]

Supplementary material for

Increased elasticity of melanoma cells after low-LET proton beam due to actin cytoskeleton rearrangements

Katarzyna Jasińska-Konior, Olga Wiecheć, Michał Sarna, Agnieszka Panek, Jan Swakoń, Marta Michalik, Krystyna Urbańska, Martyna Elas

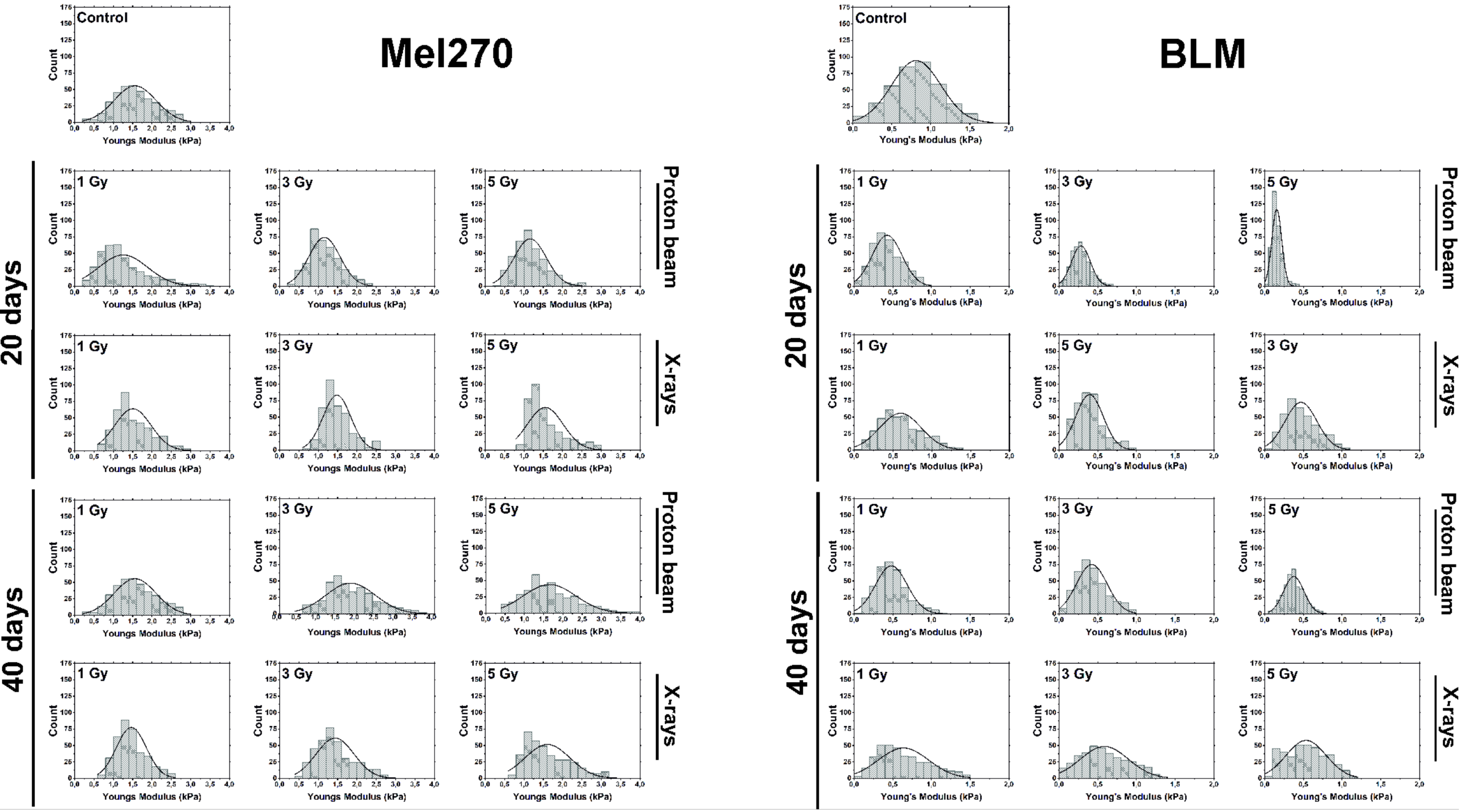

Fig. A1. The influence of proton beam radiation and X-rays on the elastic properties of Mel270 and BLM cells. (A) Histograms of Young’s modulus values for Mel270 cells; (B) Histograms of Young’s modulus values for BLM cells.

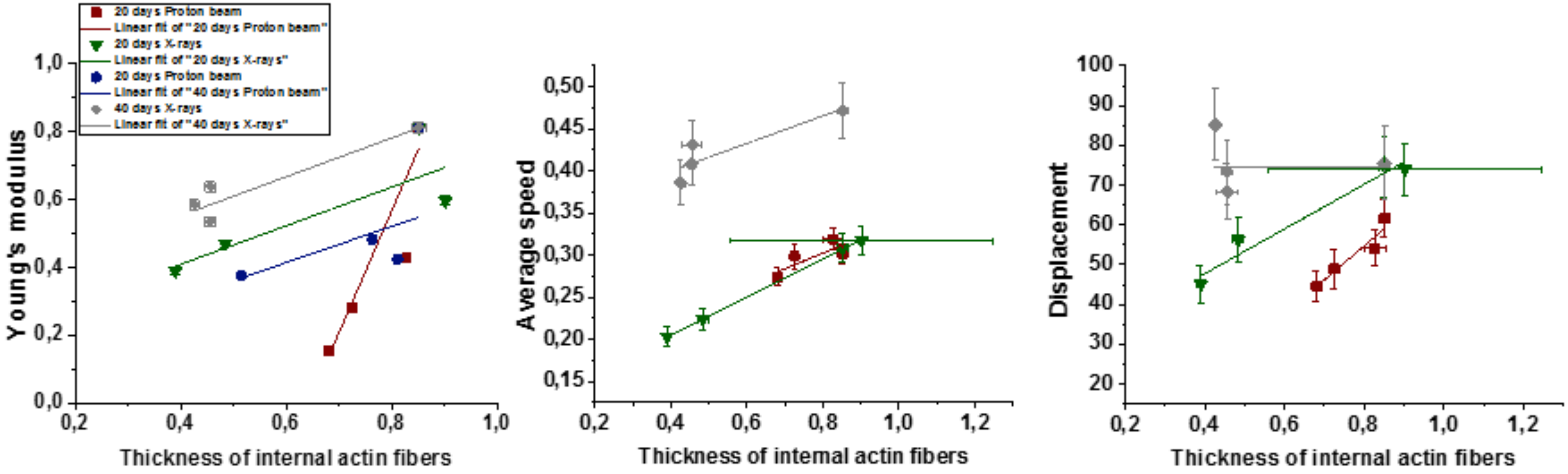

Fig. A.2. The thickness of internal actin fibers decreased in BLM cells treated with both types of radiation. This decrease was accompanied with a decrease in Young modulus (i.e. increase in elasticity of cells), as well as with a decrease in migration parameters, such as distance and mean rate of displacement for both types of radiation. Due to small number of points (4 per group) the correlations could not be statistically significant. Migration for cells treated with proton beam and observed 40 days later was too low and excluded from consideration.

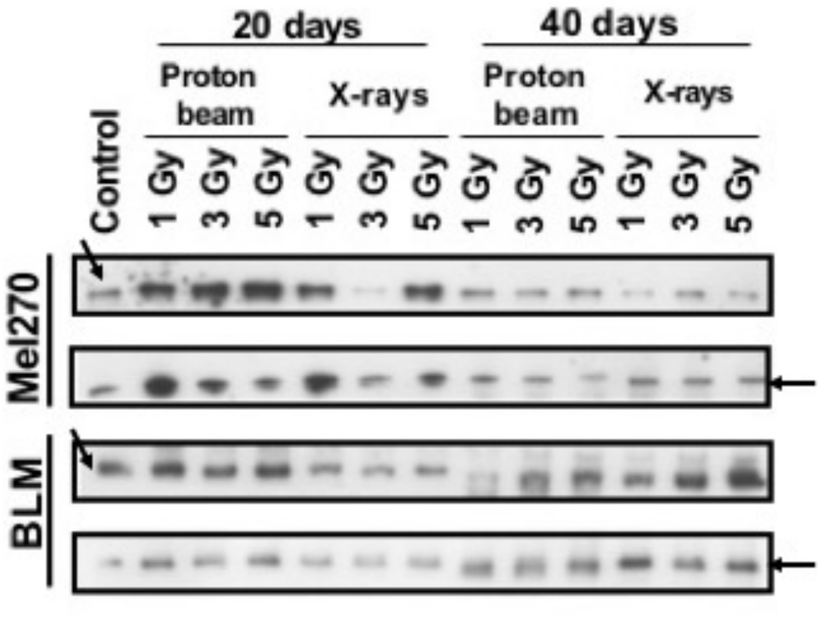

Fig. A.3 Western blot results for TNC from Fig. 6. Protein expression determined with Western Blot in Mel270 and BLM cells after treatment with different doses (1, 3, 5 Gy) of proton beam or X rays. Cells were lysed 20 and 40 days after irradiation. Arrows show control for TNC was taken from the upper blot and all the other samples were taken from the lower blot.
